# Zika Virus Neuropathogenesis—Research and Understanding

**DOI:** 10.3390/pathogens13070555

**Published:** 2024-07-02

**Authors:** Anna D. Metzler, Hengli Tang

**Affiliations:** Department of Biological Science, Florida State University, Tallahassee, FL 32306, USA

**Keywords:** Zika virus, neuropathogenesis, neuronal apoptosis, microcephaly, Guillain-Barré Syndrome, cell cycle dysregulation, organoid models, animal models, blood–brain barrier

## Abstract

Zika virus (ZIKV), a mosquito-borne flavivirus, is prominently associated with microcephaly in babies born to infected mothers as well as Guillain-Barré Syndrome in adults. Each cell type infected by ZIKV—neuronal cells (radial glial cells, neuronal progenitor cells, astrocytes, microglia cells, and glioblastoma stem cells) and non-neuronal cells (primary fibroblasts, epidermal keratinocytes, dendritic cells, monocytes, macrophages, and Sertoli cells)—displays its own characteristic changes to their cell physiology and has various impacts on disease. Here, we provide an in-depth review of the ZIKV life cycle and its cellular targets, and discuss the current knowledge of how infections cause neuropathologies, as well as what approaches researchers are currently taking to further advance such knowledge. A key aspect of ZIKV neuropathogenesis is virus-induced neuronal apoptosis via numerous mechanisms including cell cycle dysregulation, mitochondrial fragmentation, ER stress, and the unfolded protein response. These, in turn, result in the activation of p53-mediated intrinsic cell death pathways. A full spectrum of infection models including stem cells and co-cultures, transwells to simulate blood–tissue barriers, brain-region-specific organoids, and animal models have been developed for ZIKV research.

## 1. Zika Virus and Its Replication

Zika virus (ZIKV) is a flavivirus of the Flaviviridae family. Its main route of transmission is via the Aedes aegypti mosquito [1]. Other transmission routes, such as mother-to-child, sexual, and blood-borne, have also been reported [1]. A ZIKV infection is usually asymptomatic. However, specific ZIKV strains that have recently emerged are associated with neurological diseases such as encephalitis and Guillain-Barré Syndrome (GBS) [2,3]. In the event of maternal ZIKV infection during pregnancy, the risk for the development of microcephaly and congenital fetal malformations is increased [4]. The mechanisms by which ZIKV causes these diseases have not been fully understood. In this review, we will explore the infection and effects of ZIKV in the body, especially the impact on the nervous system. We will also discuss recent advancements in both laboratory research (in vivo and in vitro) and clinical studies that have significantly contributed to our understanding of the neurological diseases caused by ZIKV. 

### 1.1. ZIKV Genome and Proteins

While some flaviviruses such as DENV have multiple serotypes [5], ZIKV only forms one serotype with two main lineages: an African lineage and an Asian lineage. MR766 is the original African strain isolated in 1947 from the Ziika forest in Uganda [6]. Other African strains include ZIKV-MP1751 (Uganda, 1962) and Dakar 41525 (Senegal, 1984). The most recent outbreaks observed, which were linked to more severe diseases around 2015-2016 in South America, were caused by viruses within the Asian lineage such as PRVABC-59 (Puerto Rico, 2015), FB-GWUH-2016 (Guatemala, 2016), MEX1-44 (Mexico, 2016), and SZ01 (Samoa/Shenzhen, 2016). Related earlier Asian isolates include FSS13025 (Cambodia, 2010), H/PF/2013 (French Polynesia, 2013), Haiti/1225/2014 (Haiti, 2014), and p6-740 (Malaysia, 1966) [7].

**Figure 1:**
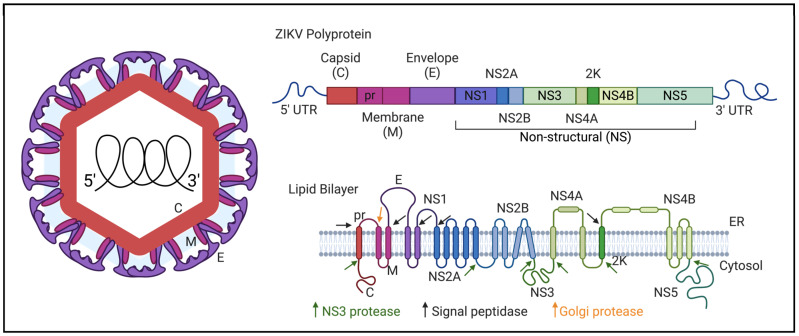
ZIKV virion, genome, and polyproteins. The topology of the viral proteins in the ER membrane as well as sites of protease processing are shown. Green arrows indicate viral NS3 protease cleavage sites; black and orange indicate host protease sites (Created with BioRender.com, accessed on 1 May 2024).

ZIKV contains a 10.7kb positive-sense single-strand RNA (ssRNA) genome, which encodes three structural and seven nonstructural (NS) proteins. Following the 5’ noncoding region, the structural genes encode capsid (C), a precursor peptide linked to the membrane protein (prM), and an envelope (E) protein. They are followed by the nonstructural genes NS1, NS2A, NS2B, NS3, NS4A, NS4B, and NS5, and, lastly, the noncoding region at the 3’ end without a poly(A) tail [8,9]. The genome is translated into one single polyprotein which is cleaved by host and viral proteases to produce the discrete viral proteins (Figure 1). 

The functions of each protein have been described extensively. Overall, the structural proteins are involved in virion assembly, absorption, and cell entry, while the NS proteins facilitate the replication and translation of the viral genome, as well as the regulation of the host immune response [10]. A more specific description of each protein’s function in the replication cycle can be found in Table 1.

### 1.2. ZIKV Replication

**Figure 2:**
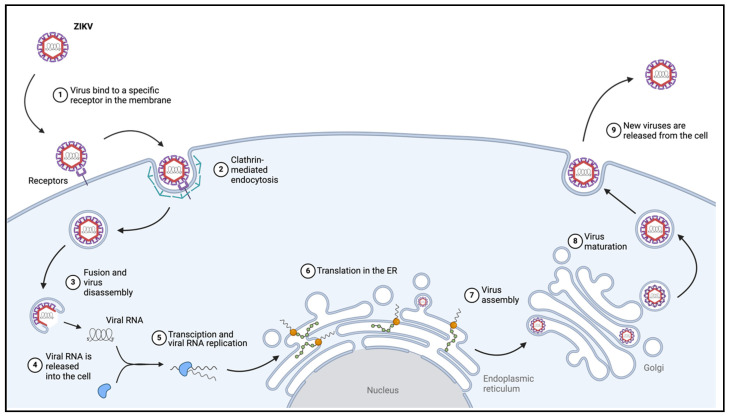
Generalized ZIKV infection cycle (Created with BioRender.com, accessed on 1 May 2024).

The life stages of ZIKV infection follow that of the typical replication cycle of a positive-strand RNA virus which includes viral entry, the translation and replication of the viral genome, the production of the new virus, and, lastly, the maturation and release of new virions from the host cell (Figure 2).

To enter the cell, the virion attaches to the target cell surface via an interaction between the viral envelope (E) protein and specific cellular receptors. Dendritic cell-specific intracellular adhesion molecule 3-grabbing nonintegrin (DC-SIGN) [22], the phosphatidyl serine (PS) receptor proteins AXL [23], neural cell adhesion molecule (NCAM1) [24], heat shock protein 70 (HSP70) [25], and integrin αvβ5 [26] are examples of reported entry receptors. The function of AXL and related PS receptors in ZIKV entry is highly controversial. Of the many publications addressing this question, there is an approximately even split between yes and no in terms of the answer [23,27,28,29,30,31,32,33,34,35,36,37,38,39,40,41,42,43,44,45,46]. Even studies using definitive ablation methods such as gene knockout came up with opposite conclusions. Moreover, among the studies showing a positive result for the PS receptors’ involvement in flavivirus infection, there is disagreement on the mechanism of action, with hypotheses ranging from PS-mediated attachment/entry [33,47,48,49] to the suppression of the antiviral interferon (IFN) response [23,39,40,50]. 

Virion binding to the cell surface is followed by clathrin-mediated endocytosis, leading to the formation of an endosome containing the virus [51,52]. The acidification of the endosome triggers the fusion of the endosomal and viral membrane, resulting in the release of the viral genome into the cytoplasm. Next, the positive-sense RNA genome is translated into the polyprotein, the cleaving of which releases the structural and NS viral proteins. In addition, the genome is transcribed into a negative-sense RNA, which serves as the template for RNA replication carried out by the viral RNA-dependent RNA polymerase NS5 and assisted by the additional NS proteins. The production of viral particles occurs within structurally distinct replication organelles along the ER cisternae [53]. As the ZIKV proteins move through the trans-Golgi network, viral assembly and maturation occurs. During maturation, the pr peptide is cleaved from the membrane protein, transforming the viral outer membrane from an immature rough structure to the mature smooth icosahedral shape [54,55,56]. This process is thought to be mediated by a cleavage site that is recognized by furin or furin-like proteases residing in the Golgi complex [57]. Lastly, newly produced viruses are released from the cell via exocytosis.

## 2. Cells Infected by ZIKV

ZIKV has been shown to infect and replicate in human skin fibroblasts, keratinocytes, monocytes, macrophages, and endothelial cells, as well as neuronal cells such as neuronal progenitor cells (NPCs) and radial glial cells. Each cell type displays unique infection characteristics which underlie the broad tissue tropism and disease development associated with ZIKV.

### 2.1. Non-Neuronal Cells Infected by ZIKV

**Figure 3:**
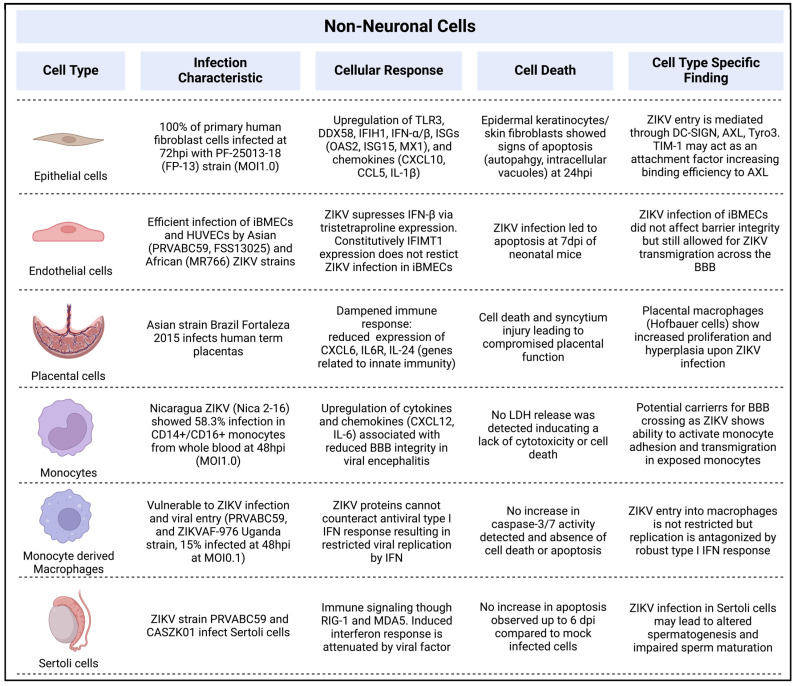
Overview of non-neuronal cells infected by ZIKV and notable infection characteristics, cellular response, cell death, and cell-type-specific findings [27,33,40,58,59,61,62,66,67,68,70,72,73,74,75] (Created with BioRender.com, accessed on 1 May 2024).

Among the non-neuronal cells susceptible to ZIKV infection are skin cells, placental cells, blood cells, and Sertoli cells (Figure 3). For instance, in the skin, primary fibroblasts, epidermal keratinocytes, and dendritic cells have been demonstrated to be susceptible to ZIKV infection [58]. In blood, the main targets for ZIKV are monocytes and macrophages [59,60]. Ayala-Nunez et al. reported that ZIKV triggers the activation of monocytes’ adhesive properties and facilitates their transmigration across the blood–brain barrier (BBB) [61], suggesting that monocytes may act as carriers for ZIKV entry into the brain. Additionally, macrophages have been found to be vulnerable to ZIKV infection [62,63,64], although viral replication is limited due to the virus’ inability to counteract STAT1/STAT2 phosphorylation and the antiviral interferon response [62]. Endothelial cells, specifically brain microvasculature endothelial cells (BMECs), form the BBB and are supported by astrocytes and pericytes [65]. ZIKV (PRVABC59 or MR766) has been shown to infect BMECs and cross the BBB without disrupting barrier function [66,67]. The involvement of suppressed IFN-β signaling and the suppression of IFITM1 are correlated with ZIKV infection in BMECs [66,68]. Similarly, human umbilical vein endothelial cells can be infected by ZIKV [27,33]. The susceptibility of placental cells to ZIKV infection varies in vitro [69,70,71], potentially due to the secretion of type III interferon [71] and the different responses to them in the different in vitro systems used [69,70,71]. Furthermore, Hofbauer cells (placental stromal macrophages) exhibit increased proliferation and hyperplasia following ZIKV infection [72]. Sertoli cells, the barrier and immune cells found within testes, are readily infected by ZIKV [73] and show a robust immune response signaling through RIG-I and MDA5 signaling [74]. However, bone morphogenic protein (BMP6) signaling in Sertoli cells was suppressed by ZIKV, impairing the BMP6-dependent increase in IFN-β, p-IRF3, and p-STAT1 levels. The infection of Sertoli cells has been connected to altered spermatogenesis in mice [75].

### 2.2. Cells of the Nervous System That Are Infected by ZIKV

With a clear association between ZIKV and neurological disorders, particularly microcephaly in newborns, it is crucial that we understand the impact of ZIKV on brain development and the underlying factors that may contribute to the onset of microcephaly. The nervous tissue consists of neurons and neuroglia, with the latter supporting neuronal cells in their functions. Throughout neuronal development, neural cells undergo maturation and migration from the ventricular zone (VZ) towards the cerebral cortex through the subventricular zone (SVZ) and intermediate zone, presenting many opportunities for ZIKV to impact development.

#### 2.2.1. Radial Glial Cells

Radial glial cells are the primary cells that originate from the neuroepithelium during the process of neuronal development and are responsible for the development of all neurons found in the mature brain [76]. Initially, these cells localize across the VZ and migrate towards the cortical plate. Wu et al. (2016) showed that infection of pregnant mice with ZIKV resulted in infection of radial glial cells in the VZ of the fetus [77]. This viral infection subsequently leads to a reduction in the proliferation of cortical neural progenitor cells, ultimately causing abnormalities in the brain development of the offspring mice [77]. 

#### 2.2.2. Neuronal Progenitor Cells

NPCs differentiate from radial glial cells by asymmetric division [78]. Each division produces a self-renewed radial glial cell and an NPC. Dividing NPCs are also known as basal progenitor cells [79] or intermediate progenitor cells [80]. These cells are predominantly located in the SVZ and play a crucial role in embryonic brain development. The disruption of this process due to ZIKV infection can have severe consequences on neuronal development and has been proposed as a primary factor contributing to microcephaly. Accordingly, ZIKV infection has been linked to cell cycle arrest and attenuated growth in human neuronal progenitor cells (hNPCs) [81,82]. It was also reported that the cell cycle is prolonged in infected NPCs as compared to that in uninfected cells, and infected NPCs experienced neuronal death and axonal rarefaction [83]. These data indicate that ZIKV infection disrupts the development and maturation of NPCs, which can lead to cortical thinning. ZIKV also exhibits strain-specific infection patterns in hNPCs. The African ZIKV stain (MR766) infects hNPCs at a higher rate (69.8% at MOI 0.02) compared to the Asian strain (FSS13025) (46.7% at MOI 0.04) at 64 h.p.i. [84]; whether this strain-specific difference is related to neuro-disease severity is currently unknown. 

#### 2.2.3. Astrocytes

Astrocytes, also known as astroglia, are star-shaped glial cells present in the brain and spinal cord. They represent one of the most common cell types in the brain and play various dynamic roles such as the secretion or absorption of neural transmitters and the maintenance of the BBB through biochemical regulation of the endothelial cells forming the BBB. Astrocytes are susceptible to neurotropic flavivirus infection [85]. ZIKV replication in astrocytes was detected in mice brains [86] and more recent in vitro studies have shown that primary human astrocytes remain infected and shed virus for over a month [87,88]. This prolonged infection may play a crucial role in causing neuronal damage, as evidenced by the dysregulation of genes involved in the morphogenesis of the epithelium, adherens junctions, and focal adhesions [87]. Furthermore, during ZIKV infection, a limited cytokine response has been observed. The cytokine response to infection with MR766 or PRVABC59 is mainly limited to CXCL10, IL-6/8/12, and CCL5 [89,90]. This immune response correlates with significant structural alterations in the cells. Electron tomography revealed an increase in small vacuoles containing neurosecretory vesicles and collapsed endoplasmic reticulum cisternae, indicating extensive cellular remodeling following infection [89]. Live cell imaging further revealed increases in the mobility of vesicles upon ZIKV infection [91].

#### 2.2.4. Microglia

Microglia, the primary immune cells of the central nervous system (CNS), are resident macrophages that are distributed throughout the brain and spinal cord. Originating from yolk-sac progenitors, these cells migrate into the CNS during early development, before the closure of the BBB [92]. Microglia are sustained through local proliferation and function to maintain homeostasis and defend against pathogens. However, they have also been implicated as potential carriers of ZIKV during vertical transmission in early pregnancy, which can contribute to the development of microcephaly. This is particularly significant as the biogenesis of microglia occurs in close proximity to the maternal vasculature [93]. It has been hypothesized that invading microglia could potentially carry ZIKV. The virus released from the microglia could infect immature neuronal stem cells during the early stages of pregnancy, even before the initiation of angiogenesis in the developing brain [93]. While most neuronal cells display signs of cell death upon infection, a relatively high viral load is required to infect microglia (up to a multiplicity of infection of 10) without inducing apoptosis [94,95,96]. Such prolonged infection may facilitate viral dissemination. In addition to releasing the virus to the surrounding environment, microglia induce a robust proinflammatory response upon infection through the expression of inflammatory molecules such as IFN-α, IFN-β, TNF-α, IL-1β, IL-6, MCP-1, NO, and iNOS. This has been shown with the Asian lineage ZIKV-H/FP/2013 and African lineage ZIKV-MP1751 infection [95], as well as the Asian lineage ZIKV strain SZ01 [97] and ZIKV stain MEX1-44 [83]. The release of the inflammatory molecules from microglia may impact surrounding neurons. The increased expression of NO and iNOS from microglia has been associated with NO-mediated neuronal cell death [98]. Furthermore, flavivirus infection leads to a shift in the polarization of microglia towards the proinflammatory M1 type, which promotes inflammation and neurotoxicity within the CNS [99]. While microglia overall play a vital role in immune response and maintaining homeostasis in the CNS, they can also serve as carriers of pathogens like ZIKV, potentially causing severe consequences once infected.

#### 2.2.5. Glioblastoma Stem Cells

In addition to the healthy neuronal cells present in the brain, glioblastoma stem cells (GSCs) from patients have been found to be highly permissive to ZIKV [100]. ZIKV infects glioblastoma stem cells in a manner that relies on the presence of SOX2, leading to a decrease in tumor growth through the induction of apoptotic cell death. Furthermore, SOX2 plays a role in facilitating ZIKV infection by suppressing the innate immune response. When glioblastoma stem cell organoids were infected with ZIKV, it resulted in the upregulation of genes associated with the interferon response, programmed cell death, TLR signaling, and, notably, inflammasome signaling [26]. Findings suggest that ZIKV could be utilized as an oncolytic virus for the targeting of glioblastoma [101,102]. There are two goals in the use of ZIKV as an oncolytic virus for cancer treatment. Firstly, ZIKV can cross the BBB, allowing it to enter the brain which conventional drugs are prohibited/prevented from by the barrier. Secondly, ZIKV preferentially killed GSCs over NPCs and neuronal cells upon infection [26]. This was supported by studies in mice and rats where induced brain tumors shrank upon intracranial ZIKV (H/PF/2013) injection [103]. Additionally, mice inoculated with ZIKV (MR766 or PE243) intravenously showed detectable levels of viral RNA in the brain tissue while preserving BBB integrity initially. Upon prolonged infection and increased inflammation, cytopathic effects were observed, potentially leading to slight disruptions of the BBB over time [104].

**Figure 4:**
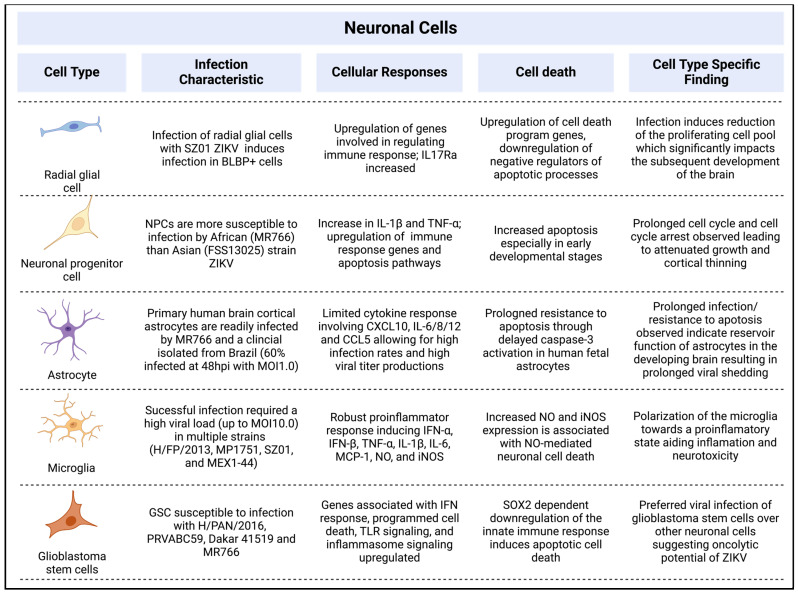
Overviews of neuronal cells infected by ZIKV and notable infection characteristics, immune response, apoptosis, and cell-type-specific findings [26,77,81,82,83,84,87,88,89,90,94,95,96,97,98,99,100,101,102,103] (Created with BioRender.com, accessed on 1 May 2024).

In summary, the diverse range of cells that ZIKV can infect underscores the complexity of its pathogenicity and the need for further research in this area (Figure 4). 

## 3. ZIKV Neuropathogenesis—Neuronal Diseases

Neuropathogenesis refers to the process by which diseases or disorders affect the nervous system, leading to the development and subsequent progression of neurological symptoms. Numerous conditions that can negatively impact the nervous system include neurodegenerative diseases (Alzheimer’s and Parkinson’s disease), infections (meningitis or encephalitis), brain tumors, and autoimmune disorders (multiple sclerosis). Viruses that target the nervous system are classified as neurotropic viruses. Of the Flavivirus genus, ZIKV, Japanese encephalitis virus (JEV), West Nile virus (WNV), and Tick-born encephalitis virus (TBEV) are a few examples of neurotropic viruses [105]. 

### 3.1. Neuronal Development, Congenital ZIKV Syndrome, and Microcephaly

A prominent manifestation resulting from ZIKV infection is microcephaly which is characterized by an abnormally small head circumference. During the ZIKV outbreak in South America in 2015/16, a considerable number of cases of microcephaly associated with ZIKV were reported. These cases involved pregnant mothers who had contracted ZIKV during pregnancy and subsequently gave birth to infants with microcephaly [3,106]. The risk of microcephaly was particularly high when the infection occurred early in pregnancy [107,108]. A case–control study from Brazil determined the association between ZIKV infection during pregnancy and the development of microcephaly in infants to be significant [109]. ZIKV-induced neurodevelopmental disorder is associated with deficiencies in brain development due to the improper differentiation of specific cells, cortical thinning, and neuronal cell death. It has been proposed that ZIKV has the ability to cross the placental barrier and infect neuronal progenitor cells, disrupting their proliferation and differentiation, ultimately leading to impaired brain growth, accompanied by abnormal skull formation [110,111]. Additionally, other abnormalities commonly observed in congenital ZIKV syndrome include calcifications primarily at the cortico-subcortical junction of the white matter, cortical malformations, ventriculomegaly (dilated lateral ventricles), cerebellar hypoplasia (reduced cerebellar volume), and corpus callosum dysgenesis [107,112]. Less commonly encountered were lissencephaly (“smooth brain”) and pachygyria (aberrations in cerebral convolutions) [113]. 

**Figure 5:**
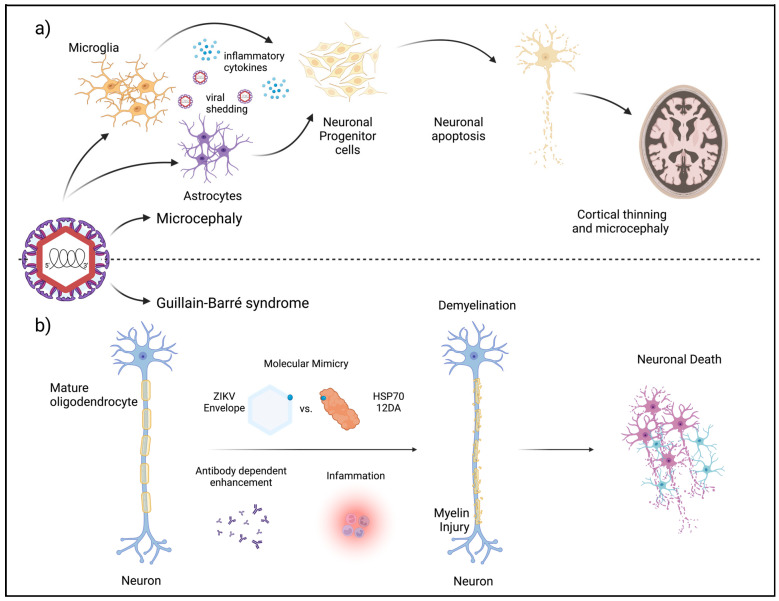
Factors in the development of microcephaly and Guillain-Barré Syndrome (GBS). (**a**) Prolonged infection of microglia and astrocytes results in the release of inflammatory cytokines and viral shedding that enhances apoptosis in neuronal progenitor cells leading to cortical thinning and microcephaly. (**b**) Antibody-dependent enhancement and molecular mimicry are two major contributors to ZIKV-induced demyelination (Created with BioRender.com, accessed on 1 May 2024).

While neuronal cells, especially neuronal progenitor cells, are quite susceptible to ZIKV-induced apoptosis, astrocytes and Sertoli cells are not as likely to undergo apoptosis upon infection. Nevertheless, ZIKV infection of astrocytes can impact neuronal cell death indirectly. Astrocytes can be infected but a majority of the infected cells, regardless of virus strain, remain resistant to apoptosis and can shed the virus up to 28 days post-infection despite a strong anti-viral response [87]. During this prolonged shedding period, an increase in neuronal apoptosis surrounding the infected astrocytes was observed. This indicates that astrocytes could act as a reservoir for ZIKV within the CNS [114]. This observation has also been reported in brain tissue from a 20-week gestation fetus with a confirmed ZIKV infection [115]. Similar results have also been seen in the settings of DENV [116] and WNV infection [117]. This type of bystander apoptosis can be a result of either virus-shedding or cytokine secretion from infected neighboring cells such as astrocytes or microglia [94,118] (Figure 5a). 

### 3.2. Guillain-Barré Syndrome in Adults

In addition to neurological complications in infants and negative outcomes during pregnancy, ZIKV has also been linked to the development of GBS in adults. GBS is characterized by a sudden onset of muscle weakness and ascending paralysis from the immune system’s attack on the peripheral nervous system. During the ZIKV outbreaks in Latin America and French Polynesia, an increase of up to 9.8-fold in GBS was reported [119,120,121]. Antibody-dependent enhancement (ADE) and molecular mimicry are among the proposed mechanisms for ZIKV-associated GBS development (Figure 5b). ADE describes a phenomenon observed in viral infection where pre-existing antibodies bind to a virus without effectively neutralizing it. Conversely, these antibodies facilitate viral entry into host cells through interactions with Fc receptors on immune cells, thereby enhancing viral infectivity [122]. Pre-existing and elevated levels of antibodies to DENV and ZIKV have been found in the sera of patients with ZIKV-associated GBS [123,124]. Additionally, an analysis of anti-DENV monoclonal antibodies revealed that a majority of them also reacted with ZIKV [123]. Aligning with this information, most patients showing ZIKV-associated GBS had evidence of prior DENV infection, suggesting a potential synergistic effect between ZIKV and pre-existing DENV immunity in triggering GBS [125]. On the other hand, the presence of other infections like Mycoplasma pneumoniae in individuals with ZIKV can exacerbate GBS development through immune dysregulation [125,126]. In addition to compounding infections, ZIKV may also enhance the production of certain autoimmune antibodies relevant to GBS. For example, an increase in anti-ganglioside IgG and IgM antibodies was detected in ZIKV patients with GBS [127,128]. Moreover, the presence of shared immunological epitopes between ZIKV and human proteins associated with demyelination and axonal neuropathy suggests a role of molecular mimicry in the development of GBS upon ZIKV infection [129]. Specifically, a glycan loop (GL) region of the envelope protein contains an IVNDT motif. This motif is conserved in two human neuronal proteins, Heat Shock 70 kDa protein 12A (HSP70 12A) and voltage-dependent L-type calcium channel subunit alpha-1C (Cav1.2) [130]. 

CNS invasion by ZIKV can trigger neuroinflammatory responses that result in neuronal damage and peripheral neuropathy [131]. Variations in the ZIKV genome, particularly in the NS1 gene, may enhance its neurovirulence and ability to evade the immune system, increasing the risk of GBS [132,133]. The virus’s persistence in the CNS, supported by pro-inflammatory and anti-apoptotic pathways, worsens neurological complications including GBS [134]. ZIKV has also been shown to directly infect human peripheral neurons and Schwann cells, leading to substantial cell death [135,136]. NS1 has been suggested to elicit neutrophil extracellular traps induced by the upregulation of CXCL1 and IL-1β as well as the activation of caspase 3. These alterations may injure the peripheral nervous system [137]. These findings collectively emphasize the complex interplay between viral infection, host immunity, and neurological complications, thus necessitating further investigation into the precise mechanisms driving GBS in the context of ZIKV infection.

## 4. ZIKV Neuropathogenesis—Cellular Mechanisms

### 4.1. Cell Cycle Perturbation and Mitotic Catastrophe

ZIKV replication in infected cells has been shown to induce DNA damage by causing double-stranded breaks (DSBs) in the host genome [82,138]. These DSBs activate the DNA damage response (DDR) pathway through ATM/Chk2 signaling. The DSBs can be detected with an increase in γH2Ax observed in infected cells [82,138,139], which recruits p53-binding protein 1 (53BP1) and activates the p53 pathways. The expression of p53 has been shown to be upregulated in ZIKV infection with PRVABC59, which limits cell growth via p21/PUMA [139]. The ATR/Chk1 signaling pathway is not activated by ZIKV [82,140], indicating that single-stranded breaks are not induced upon ZIKV infection. Along with the activation of ATM/Chk2, CDC25 phosphorylation, and cyclin A and cyclin E prevent neuronal progenitor cells from progressing through S-phase, blocking successful DNA replication. The arrest of NPCs in S-phase via the drugs Aphidicolin or Thymidine has been shown to increase ZIKV replication [82]. However, the specific ZIKV proteins that are capable of inducing this DNA damage remain unknown. 

Unresolved DNA damage induced by ZIKV infection also impacts mitosis. ZIKV has been found to induce mitotic catastrophe in hNPCs [140]. Rychlowska et al. identified that this catastrophe is triggered by mitotic entry in the presence of DNA damage, due to the ZIKV-mediated depletion of nuclear polynucleotide 5′-kinase 3′-phosphatase (PNKP). PNKP is a critical DNA damage repair enzyme that has been found to relocate into the cytoplasm together with NS1 upon ZIKV infection. They further report that ZIKV can activate the cytoplasmic CycA/CDK1 complex, which triggers an unscheduled mitotic entry despite DNA damage [140]. This mitotic catastrophe was also observed when the Envelop protein was overexpressed in neuronal crest cells PC12 cells [141]. As inhibitors of caspase-3 and caspase-9, not caspase-8, could block the apoptosis in these cells, ZIKV likely triggers the apoptosis in these cells via an intrinsic cell death signaling pathway. Additionally, ZIKV infection may affect the recruitment of centrosomal proteins and has been suggested to result in centrosomal structural defects. This affects the symmetric division of NPCs, potentially depleting the NPC pool and, ultimately, impairing VZ development [142].

### 4.2. Mitochondrial Fragmentation

Mitochondrial fragmentation correlated to apoptosis was previously observed in cells infected by other flaviviruses. The overexpression of DENV proteins has been shown to alter mitochondrial bioenergetics, leading to changes in the mitochondrial membrane potential, and ultrastructural alterations such as mitochondrial swelling and membrane blebs [143,144]. These are characteristic changes seen in cells undergoing apoptosis [145]. ZIKV infection in neuronal stem cells (hNSCs) and glioblastoma cells (SNB-19) disrupts mitochondrial dynamics through a decrease in MFN2 protein levels, leading to changes in the mitochondrial network structure that may contribute to ZIKV-mediated neuronal cell death [146]. It has been found that ZIKV infection induces the conformational activation of Bax in the mitochondria and the subsequent activation of caspase 3 [147]. The reduction in Bax expression inhibited the cytochrome C release from the mitochondria and preserved the mitochondrial membrane potential, which has been found to decrease upon ZIKV infection. The knockdown of Bax resulted in decreased cell apoptosis in the neuroblastoma cell line SH-SY5Y cells upon ZIKV infection [147].

### 4.3. Endoplasmic Reticulum Stress and Unfolded Protein Response

During ZIKV infection, viral replication and translation occur on the endoplasmic reticulum (ER) membrane. The remodeling of the ER membrane and localization of all viral proteins to the ER facilitate efficient replication [18,148]. The ZIKV-induced remodeling of the ER has been observed in diverse cell types derived from humans (Huh7, hNPCs, SK-N-SH, and HeLa), primates (Vero), and mosquitos (C6/36) [149,150,151,152]. These structural modifications create a protective environment for viral genome replication, allowing for optimized viral replication. The expression of viral proteins increases the protein folding demand and activates ER stress sensor transmembrane proteins (PERK, ATF6, and IRE1), stimulating the unfolded protein response (UPR) [150,153,154,155]. The UPR is a conserved mechanism which resolves and facilitates proper protein folding in the ER by upregulating the expression of chaperone proteins such as GRP78, calnexin, calreticulin, and protein disulfide isomerase (PDI) [150,153,154]. 

If the UPR response is unsuccessful in restoring ER homeostasis and proper protein folding, prolonged ER stress can occur. Prolonged ER stress due to continuing viral replication has been linked to the formation of stress granules (SGs), which negatively impact viral genome replication [156]. Accordingly, ZIKV has been found to suppress the formation of SGs through the upregulation of hosts Growth Arrest and DNA-Damage-inducible 34 (GADD34) protein [153]. The inhibition of GADD34, in turn, suppresses ZIKV replication [157]. The viral proteins capsid, NS3/NS2B3, and NS4A have been shown to interfere with SG formation in A549 and human fetal astrocytes [158]. 

The UPR also activates the ER-specific autophagy process called reticulophagy, to target viral proteins for degradation [159]. ZIKV and Dengue virus evade reticulophagy by the NS2B3-mediated cleavage of Family with Sequence Similarity 134 Member B (FAM134B), a reticulophagy receptor protein [160]. Lastly, the total ER stress burden caused by ZIKV infection can induce paraptosis-like cell death through the PI3K/Akt signaling axis, which induces large cytoplasmic vacuoles [161].

### 4.4. Central Regulators—p53 and Caspase-3 Activation

Multiple cellular stresses—DNA damage, cell cycle arrest, mitochondrial fragmentation, and the UPR—can result in apoptosis upon ZIKV infection through the intrinsic apoptotic pathway and caspase-3 activation. The general activation of apoptosis has been shown for many ZIKV strains, although strain-specific mechanisms have been described. FSS13025, H/PF/2013 and Haiti/1225/2014 have been shown to increase p53 expression [84,141,162], which inhibits BCL-2 and leads to the activation of Bax- and caspase-3-induced apoptosis. Infections with FSS13025 resulted in less apoptosis when the cells were treated with a p53 inhibitor [84]. MR766, on the other hand, inhibits p53 and signals through the JNK pathways, activating serine 139 phosphorylation of histone H2Ax (γH2Ax), which upregulates caspase-3- and PARP-induced apoptosis in hNPCs and hNSCs [84,139]. PRVABC59 infection does not increase PARP cleavage or caspase-3 activation in hNSCs, but it upregulates the serine 15 phosphorylation of p53, leading to p21/PUMA expression which, ultimately, limits cell growth [139]. Overall, the activation of these pathways may result in a smaller cortical neuronal progenitor cell population which leads to a smaller brain size with damaged brain structures.

### 4.5. Immune Response and Neuroinflammation

The pathogenesis of ZIKV infection is also closely connected to the modulation of the host immune response, particularly interferon and inflammatory pathways. An analysis of the transcriptome in ZIKV-infected developing brains has shown a significant increase in genes related to the immune response, especially those involved in the interferon response (OASl2, USP18, IFIT1, MX2, OAS1b, IFIT3, LIGP1, DDX60, IFI44, and IRF7) [83] and cytokine production (IL-1β, TNF, CXCL10, IFN-Β1, and TLR3) [163]. MR766 triggers the activation of innate immune receptors such as TLR3, which disrupts genes related to neurodevelopment and decreases organoid volume [164]. Furthermore, ZIKV nonstructural proteins, particularly NS5, have varying effects on type I and type II interferon signaling pathways, inhibiting type I while stimulating type II interferon signaling. The NS5 protein of several ZIKV strains (PRVABC-59, MR766, and H/PF/ 2013) has been found to induce STAT2 but not STAT1 degradation, which affects the STAT1–STAT2–IRF9 complex and the subsequent activation interferon-stimulated response elements (ISREs) leading to a decrease in interferon-stimulated gene (ISG) expression [165,166]. On the other hand, the STAT1–STAT1 complex formation was actually increased by PRVABC59 infection, leading to upregulated IFN-γ stimulated genes including the pro-inflammatory cytokine CXCL10. Inhibiting the IFN-γ receptor and subsequent signaling suppressed ZIKV replication and the viral induction of Type II ISGs [166]. A summary of IFN-β induction and signaling by different viral strains and in multiple cell types is provided in Table 2.

The ZIKV-induced inflammatory response has been linked to the disruption of the BBB and subsequent neuroinflammation. ZIKV-infected endothelial cells, pericytes, and astrocytes—all are part of the BBB—show an increased expression of inflammatory cytokines (IL-6 and IL-8) and chemokines (CXCL10 and CCL5) in a human brain-like endothelial cell (hBLEC) model [171]. This inflammation, together with leukocyte recruitment [171,172] and impaired blood vessel development in the brain [173], could contribute to ZIKV neuroinvasiveness. 

In summary, the neuronal apoptosis induced by ZIKV is a result of a multifaceted interplay between viral factors and host responses. The activation of apoptotic pathways by different viral strains and the initiation of bystander apoptosis through inflammatory cascades are just some of the ways in which ZIKV infection disrupts the normal functioning of developing neuronal tissues. This disruption is further exacerbated by mitotic catastrophe, mitochondrial fragmentation, and ribosomal stress, all of which contribute to the severe cellular damage observed. Additionally, the modulation of immune responses, particularly interferon signaling, adds to the complexity of the interactions between the host and the virus in ZIKV pathogenesis. The disruption of the BBB and the occurrence of neuroinflammation further enhance the neurovirulence of ZIKV. Therefore, it is crucial to understand these highly specific mechanisms in order to develop effective therapeutic interventions and preventive strategies against the neurodevelopmental abnormalities associated with ZIKV.

## 5. Models Used for ZIKV Research—Advantages and Limitations

The research to study the mechanism described above has been conducted utilizing numerous cell types, culture modalities, or more complex systems such as 3D organoid and animal models. Each model has its advantages but also its own unique limitations that need to be considered when interpreting results. 

### 5.1. Stem Cells and Co-Culture

Cell culture models are commonly used to understand cell-specific infection mechanisms and characteristics. iPSCs-derived cells have been a critical component in studying ZIKV infection and modeling its impact on neuronal development at different stages [174]. To better understand the complex interactions among various cell types in the brain, co-culture models have been utilized. These models replicate the interplay between different cell types and their surrounding environment more accurately. Specific models used to mimic BBB have been described. Medina and Tang optimized a BBB model based on iPSCs differentiated into brain microvascular endothelial cells [175,176], whereas Clé et al. utilized human brain-like endothelial cells (hBLECs) from human umbilical cord blood [171]. Both models involve the differentiation of cells into BBB-like endothelial cells and the culturing of neuronal cells or astrocytes on the other side of a transwell. It has been shown that ZIKV is capable of crossing the barrier and infecting cells such as astrocytes in the lower chamber of a transwell [66,171]. Although these studies provided significant insights into potential mechanisms by which ZIKV invades the CNS, the models are still limited by the number of cell types included.

### 5.2. 3D Brain Organoid Models

The ability of 3D brain organoids, derived from PSCs using a method pioneered by Lancaster et al. [177], to model early brain development made it a clear choice for many labs in the race to uncover the cellular tropism and developmental impact of ZIKV infection on the human brain [8,178]. The initial publication reporting that ZIKV efficiently infects neural progenitor cells and induces cell death in monolayers [81] was quickly followed by a wave of brain organoid studies from many groups [164,179,180,181]. Since then, many ZIKV isolates and organoids of various stages of maturation have been used in combination. For a detailed technical summary of the major ZIKV/brain organoid studies, the readers are referred to an excellent recent review [182]. The infection studies with brain organoids confirmed the preferential targeting of SOX2+ neural progenitors [142,164,179,181,183], which are enriched in the ventricular zone (VZ) and subventricular zone (SVZ) in brain organoids. Consistent with this finding, the detection of the ZIKV antigen in VZ and SVZ is often stronger over other parts of the brain in infected fetal or embryonic mouse brain tissue [77,163,184,185], and the depletion of neural progenitors has been observed in experimentally infected non-human primate model [186,187,188]. Other congruent ZIKV phenotypes from brain organoid studies include increased cell death, thinner cortical layers, and overall reduced organoid size [29,142,164,179,181,183,189,190,191]. In addition to the cell cycle arrest of NPCs, mechanisms such as the disruption of radial glial scaffolding, the upregulation of TLR3 expression, or the induction of autophagy can all contribute to the overall reduction in brain organoid growth [164,192,193]. 

Brain organoids have also been used to evaluate potential anti-ZIKV compounds for therapeutic development [183,190,194,195,196]. The complexity and the relatively low throughput of the system make them better suited for confirmation instead of primary screening steps of compound identification studies.

### 5.3. Mouse Models

Several mouse models have been developed and have provided valuable insights into the mechanisms underlying ZIKV-induced neurologic disorders and prenatal complications. These models can be grouped into four categories: interferon knockout (KO) models, models utilizing monoclonal antibodies against the IFN receptors, mice expressing human STAT2, and neonatal animals which are presumably less immune-competent. 

Generally, immune-competent mice are resistant to ZIKV infection. Thus, immune-compromised mice have been utilized. Particularly, mice lacking the ability to complete the interferon signaling cascade through Ifnar1 KOs (A129 mice and Ifnar1^−/−^ C57BL/6 mice) or mice deficient in the interferon regulatory factors Irf3, Irf5, and Irf7 (Irf3^−/−^ Irf5^−/−^ Irf7^−/−^ triple knockout [TKO]) [197,198]. These mice developed severe disease upon infection with MR766, H/PF/2013, and Dakar strains [197]. A129 (lacking type 1 interferon response) and AG129 (lacking both type 1 and type 2 interferon response) developed both encephalitis and CNS injury upon infection with H/PF/2013, MP1751, and FSS13025 [198,199,200]. 

Additionally, a transient approach utilizing monoclonal antibodies against IFN receptors can be used to enable ZIKV infection in mice. Anti-IFNAR1 monoclonal antibodies that block receptor-binding by IFN are commonly used [201]. This approach does not require maintaining specific immune-compromised mouse colonies and is, thus, less time-intensive or cost-prohibitive.

ZIKV NS5—which degrades human STAT2—cannot degrade murine Stat2 [165], potentially explaining why immunocompetent mice are resistant to ZIKV infection and disease induction. Based on this knowledge, Gorman et al. developed a mouse model expressing human STAT2 instead of murine Stat2, resulting in productive ZIKV infection in these mice [202]. 

Lastly, neonatal mice, presumably less immune-competent, are permissive to ZIKV infection. In mice, crucial brain development stages that align with those in the third trimester of humans take place during the neonatal period, making this model relevant. CNS pathology and partial lethality were observed after the injection of 7- to 8-day old WT C57BL/6 mice with either ZIKV Dakar 41519 or ZIKV H/PF/2013 subcutaneously or intraperitoneally [34,197]. 

### 5.4. Non-Human Primate Models

Multiple non-human primates (NHPs), namely, rhesus macaques, African green monkeys, Syrian gold hamsters, and guinea pigs, have been used to study ZIKV infection. 

Rhesus macaques developed fever and viremia after subcutaneous inoculation with GZ01. The presence of the virus was detected in urine, saliva, lacrimal fluid, cerebral spinal fluid, semen, and vaginal swabs. This suggests that the virus has the ability to rapidly establish a systemic infection. [203,204,205]. The infection of rhesus macaques with ZIKV strains H/FP/2013 or PRVABC59 at early gestation resulted in fetal demise in 26% of infections [206]. Most importantly, rhesus macaques have been employed to assess the immunogenicity and effectiveness of active ZIKV immunization. This includes evaluating inactivated virus, DNA plasmid-based, and vector-based vaccines, as well as examining the protective efficacy of passive immunization against a ZIKV challenge [207,208]. The pitfalls of the rhesus macaque models include the high cost and limited number of animals. Moreover, the longer gestation period of rhesus macaques, as opposed to that of mice, prolongs experimental durations.

African green monkeys (AGMs) are also susceptible to ZIKV infection. Surveys of wild baboons and AGMs from South Africa, the Gambia, Tanzania, and Zambia revealed that up to 16% of the tested animals had been exposed to ZIKV [209]. In the lab, the subcutaneous, intravaginal, or intrarectal inoculation of AGM with ZIKV strain ArD 41525 produced viremia and viral shedding and induced virus-specific antibodies [210]. 

Syrian gold hamsters have been used to study ZIKV infection. Wild-type hamsters developed a mild disease and detectable viremia upon intraperitoneal but not subcutaneous inoculation with Senegalese (ArD 41525) and Philippines (CPC-0840) ZIKV strains [211]. To test if immunocompromised hamsters are more susceptible to ZIKV infection, STAT2-KO Syrian gold hamsters were used in another study, which showed that the subcutaneous injection of Malaysian ZIKV (P 6–740) resulted in the infection of various organs. Viral RNA and proteins were detected in the uterus, placenta, brain, spinal cord, and testicles, and infection resulted in mortalities [212].

Guinea pigs, which have previously been established as animal models for studying congenital infections and sexually transmitted diseases [213,214], are another type of NHP model for ZIKV infection. Importantly, immunocompetent guinea pigs can be infected by ZIKV and present disease. The placental structures are similar between humans and guinea pigs and their pups are born with mature CNS systems comparable to humans at birth. These unique characteristics of guinea pigs represent the advantages of this model [215]. 

Overall, the utilization of stem cells, co-cultures, and 3D organoids, as well as murine and non-human primate in vivo models have collectively contributed to the rapid and significant advancement in our understanding of ZIKV pathogenicity.

## 6. Conclusions

The severe clinical presentations of ZIKV infection, especially in infants, fueled the extensive research on this human pathogen. There is extensive interaction between the virus and the host resulting in numerous diverse molecular pathways identified in the ZIKV-dependent neuropathogenesis. Investigations have revealed potential connections between ZIKV infection and cell cycle arrest, DNA damage, mitotic catastrophes, mitochondrial fragmentation, ER stress, and the unfolded protein response. From these studies, p53 has emerged as a pivotal player in ZIKV-induced neuronal apoptosis. Lastly, the immune response to the virus has been shown to induce substantial inflammation which may be connected to increased neuroinflammation and further contribute to neuronal death. Nevertheless, how applicable the various mechanisms identified in the experimental models are to the clinical setting of ZIKV infection and disease remains unclear. And the lack of effective treatment or prevention options for ZIKV call for more research and continued investigation into antivirals and vaccines. Finally, the continuous monitoring of the neurodevelopment of infants exposed to ZIKV is crucial, as prenatal exposure can result in brain abnormalities not as prominent as microcephaly at birth, underscoring the importance of ongoing vigilance in addressing potential long-term consequences.

## Figures and Tables

**Table 1:** ZIKV viral proteins and their functions in viral replication.

	Protein	Functions and Characteristics	References
Structural proteins	Envelope (E)	Binding to cellular receptors and entry into host cells Ubiquitination of the envelope proteins by E3-ubiquitin ligase TRIM7	[11]
	Membrane (prM/M)	Pr peptide is cleaved from the membrane protein as the virus moves through the trans-Golgi network during maturation by furin (like) proteases Potentially involved in E-protein folding prior to cleavage	[12]
	Capsid (C)	Encapsulating the RNA genome and viral core assembly	[13,14]
Non-structural (NS) proteins	NS1	Forming replication compartments in the endoplasmic reticulum (ER) lumen and immune evasion	[15]
	NS2A	Dual function in viral RNA synthesis and virion assembly Recruitment of NS2B/3 complex for cleavage of C protein of polyprotein	[16]
	NS2B	Membrane-bound cofactor that stabilizes protease and helicase activity of NS3	[17]
	NS3	Serine protease (N-terminus) and RNA helicase (C-terminus) activities.	[18]
	NS4A	Membrane-bound protein that induces remodeling of the ER membrane	[19]
	NS4B	A component of the ER membrane-associated replication complex	[20]
	NS5	RNA-dependent RNA polymerase (N-terminus) and methyltransferase (C-terminus) and immune regulation of host cell	[21]

**Table 2:** Interferon beta induction by different ZIKV strains depending on cell types. Bold highlights indication effect on type I interferon response.

Cell type	Virus strain	Effect	References
A549	MR766	**Reduced** phosphorylation of JAK1 and STAT1, which ultimately reduces IFN-β and downstream ISGs by NS5	[10]
	H/PF/2013 Individual proteins	NS5 binds with STAT2 and targets it for **degradation**	[167]
	Z11060330 Individual proteins	NS2B3 **impairs** JAK-STAT pathway by degrading Jak1 and inhibition of virus-induced apoptosis NS1 and NS4B **inhibit** type I IFN production by affecting TBK1	[10]
HEK293T	PRVABC59	**Suppressed** IFN-β induction by binding to TBK1	[168]
	PRVABC59 Individual proteins	NS5 binds with STAT2 and targets it for **degradation**	[166]
	FSS13025	**Inhibits** IFN-β production via inhibition of TBK1 activity by NS2A, NS2B, and NS4B and inhibition of IRF3 by NS4A and NS5	[168]
	DAKAR 41525	**Suppressed** IFN-β induction by binding to TBK1	[168]
Dendritic cells	MR766, PRVABC59, DAKAR 41525, P6-740	High levels of IFN-β RNA transcript levels but **restricted** IFN-β protein translation	[169]
Astrocyte	MR766, PRVABC59, R103451	12-fold **increase** in IFN-β secretion compared to uninfected cells	[170]
NPCs	MEX1-44	**Increase** in IFN-β secretion levels	[83]
Glioblastoma (U87, U251, LN229)	MR766	**Reduced** IFN-β expression compared to Sendai virus (SeV)	[10]
